# *Bacteroides thetaiotaomicron* ameliorates mouse hepatic steatosis through regulating gut microbial composition, gut-liver folate and unsaturated fatty acids metabolism

**DOI:** 10.1080/19490976.2024.2304159

**Published:** 2024-01-26

**Authors:** Hu Li, Xue-Kai Wang, Mei Tang, Lei Lei, Jian-Rui Li, Han Sun, Jing Jiang, Biao Dong, Hong-Ying Li, Jian-Dong Jiang, Zong-Gen Peng

**Affiliations:** a CAMS Key Laboratory of Antiviral Drug Research, Institute of Medicinal Biotechnology, Chinese Academy of Medical Sciences & Peking Union Medical College, Beijing, China; b Key Laboratory of Biotechnology of Antibiotics, The National Health and Family Planning Commission (NHFPC), Institute of Medicinal Biotechnology, Chinese Academy of Medical Sciences & Peking Union Medical College, Beijing, China; c Beijing Key Laboratory of Antimicrobial Agents, Institute of Medicinal Biotechnology, Chinese Academy of Medical Sciences & Peking Union Medical College, Beijing, China

**Keywords:** Bacteroides thetaiotaomicron, nonalcoholic fatty liver disease, gut microbiota, folate metabolism, unsaturated fatty acids

## Abstract

Gut microbiota plays an essential role in the progression of nonalcoholic fatty liver disease (NAFLD), making the gut-liver axis a potential therapeutic strategy. Bacteroides genus, the enriched gut symbionts, has shown promise in treating fatty liver. However, further investigation is needed to identify specific beneficial Bacteroides strains for metabolic disorders in NAFLD and elucidate their underlying mechanisms. In this study, we observed a positive correlation between the abundance of *Bacteroides thetaiotaomicron* (*B. theta*) and the alleviation of metabolic syndrome in the early and end stages of NAFLD. Administration of *B. theta* to HFD-fed mice for 12 weeks reduced body weight and fat accumulation, decreased hyperlipidemia and insulin resistance, and prevented hepatic steatohepatitis and liver injury. Notably, *B. theta* did not affect these indicators in low-fat diet (LFD)-fed mice and exhibited good safety. Mechanistically, *B. theta* regulated gut microbial composition, characterized by a decreased Firmicutes/Bacteroidetes ratio in HFD-Fed mice. It also increased gut-liver folate levels and hepatic metabolites, alleviating metabolic dysfunction. Additionally, treatment with *B. theta* increased the proportion of polyunsaturated fatty acid in the mouse liver, offering a widely reported benefit for NAFLD improvement. In conclusion, this study provides evidence that *B. theta* ameliorates NAFLD by regulating gut microbial composition, enhancing gut-liver folate and unsaturated fatty acid metabolism, highlighting the therapeutic role of *B. theta* as a potential probiotic for NAFLD.

## Introduction

Nonalcoholic fatty liver disease (NAFLD) is a common liver disorder that affects approximately a quarter of the world’s population. It encompasses a disease spectrum from mild steatosis with or without inflammation to nonalcoholic steatohepatitis (NASH), which can progress to fibrosis, cirrhosis and even hepatocellular carcinoma.^1^ NAFLD is closely associated with metabolic syndromes such as obesity, type 2 diabetes, insulin resistance, and cardiovascular disease, leading to its alternative name, metabolic-associated fatty liver disease.^2^ Currently, the traditional drug development strategies for NAFLD are usually toward metabolic targets, inflammatory pathways, gut-liver axis, and antifibrotic targets.^3^ However, there are no approved drugs for treating NAFLD to date, and the primary clinical recommendation is weight loss through dietary changes and exercise. Therefore, there is an urgent need to discover effective therapeutic agents for the prevention and treatment of NAFLD.

The emerging field of intestinal microecology has shed light on the potential of regulating the gut-liver axis as a treatment for NAFLD.^4^ Numerous recent studies have identified a link between gut microbiome changes and NAFLD’s pathogenesis. Certain bacterial strains, such as *Lactobacillus*, *Bifidobacterium*, *Akkermansia muciniphila*, and *Bacteroides uniformis*, have been found to have protective effects against NAFLD in animal models.^5–10^ We previously discovered that the abundance of the Bacteroidaceae family and Bacteroides genus was associated with the alleviation of western diet (WD)/CCl_4_-induced early NASH mice model.^11^ Among these bacteria, *Bacteroides thetaiotaomicron* (*B. theta*), a Gram-negative anaerobe in the intestinal microflora of humans and mice, has shown various beneficial effects, including reinforcement of the host mucosal barrier, maintenance of immune response homeostasis, and modulation of nutrient metabolism.^12^ *B. theta*-treated mice displayed lower hepatic steatosis and triglyceride content, restored mucosal barrier function, and reduced lipopolysaccharides (LPS) translocation in experimental alcohol-related liver disease.^13^ Additionally, *B. theta* was reported to reduce diet-induced body-weight gain and adiposity in mice.^14^ However, the protective effects of *B. theta* against NAFLD, particularly against metabolic disorders in the liver, have yet to be extensively studied. Dietary fiber and gut microbial-derived metabolites exert multiple effects on the host energy metabolism by changing the intestinal environment and directly affecting various host peripheral tissues.^15^ Hence, in this study, we aimed to investigate the effect of *B. theta* and its potential mechanisms, focusing on gut microbial composition and hepatic metabolite regulation, in protecting against NAFLD-related metabolic dysfunctions induced by a high-fat diet in mice.

## Results

### B. theta abundance is positively correlated with the improvement of lipid metabolism dysfunction in NAFLD mice

**Figure 1.**
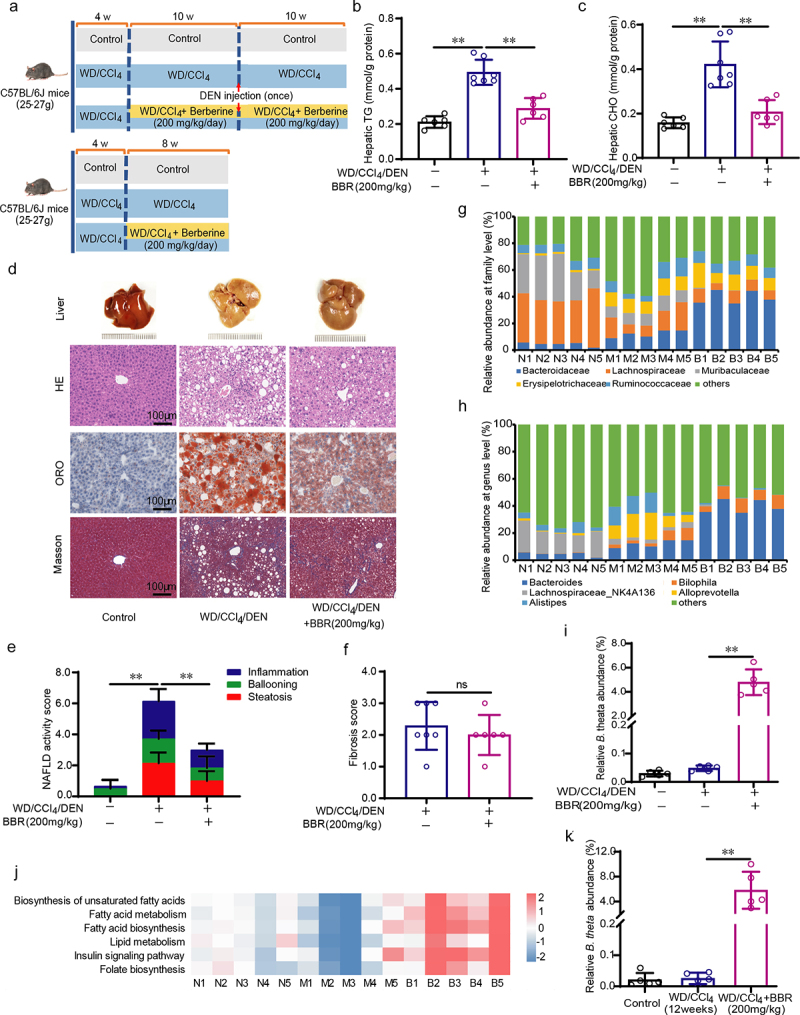
*B.Theta* abundance is positively correlated with the improvement of lipid metabolism dysfunction in NAFLD mice. (a) Schematic of the experimental design. (b, c) triglyceride (TG) (b) and cholesterol (CHO)(c) in the mouse liver (*n* = 6 ~ 7). (d) Representative liver histology visualized by H&E staining, oil red O (ORO) staining, and Masson’s trichrome staining. (e,f) NAFLD activity score (NAS) (e) and fibrosis score (f) of mouse liver (*n* = 6 ~ 7). (g-h) the abundance of the most prevalent gut microbiota at the family (g) and genus (h) levels analyzed by 16S rRNA gene sequencing with fecal samples (*n* = 5). (i) *B. theta* abundance at the species level (*n* = 5). (j) Predicted lipid metabolism-related microbiome function based on the KEGG database. (k) *B. theta* abundance at the species level in mice with WD/CCl_4_ treatment for 12 weeks. Data were presented as means with SD or represented figures. *p < .05 and **p < .01 *vs*. model or control group. ns, not significant. N, Control; M, WD/CCl_4_/DEN; B, WD/CCl_4_/DEN+BBR (200 mg/kg).

To identify the specific gut microbiota that correlated with the improvement of advanced NAFLD, which was characterized by hepatic lipid metabolism dysfunction, fibrosis, and even HCC, the mice were pre-induced by a Western diet combined with carbon tetrachloride (WD/CCl_4_) for 4 weeks. Then, we treated the mice with berberine (BBR) for 20 weeks in a WD/CCl_4_ plus diethylnitrosamine (WD/CCl_4_/DEN)−induced mouse model (Figure 1a, up) or for 8 weeks in a WD/CCl_4_−induced mouse model (Figure 1a, down) to alleviate the advanced NAFLD. As expected, BBR treatment decreased the WD/CCl_4_/DEN-induced intrahepatic TG (Figure 1b) and CHO levels (Figure 1c), ameliorated severe intrahepatic steatosis, ballooning, and inflammation (Figure 1d). However, the WD/CCl_4_/DEN-induced fibrosis was not significantly alleviated by BBR (Figure 1d). These effects were confirmed by the decreased NAFLD activity score (NAS) but not the fibrosis score (Figure 1e,f), which suggested that BBR might be more effective against metabolic abnormalities in NAFLD and less effective for the long-term and severe chemical-induced fibrosis in this experimental condition. BBR might protect against NAFLD partially through regulating intestinal microbiota.^11,16^ Intestinal microbiota sequencing analysis showed that the increased ratio of Bacteroidaceae (family) and Bacteroides (genus) was positively correlated with the improvement of advanced NAFLD (Figure 1g,h). Among the changed Bacteroides genus, *Bacteroides thetaiotaomicron* (*B. theta*) strains accounted for the highest proportion and were significantly increased by BBR (Figure 1i). Metabolic prediction through the KEGG databases indicated that these changes were associated with lipid metabolism, biosynthesis of unsaturated fatty acids, insulin signaling, and folate metabolism (Figure 1j). These results were similar to our previous report on the 8-week treatment of BBR in the early stage of NAFLD induced by WD/CCl_4_ (Figure 1a, down),^11^ which also showed an increased abundance of *B. theta* at the species level (Figure 1k) and significant alleviation of lipid metabolism dysfunction. These results suggested that a high abundance of *B. theta* might positively correlate with improving lipid metabolism dysfunction, and therapy with *B. theta* might be an effective strategy for preventing and treating hepatic lipid metabolism dysfunction in NAFLD.

### B. theta alleviates hyperlipidemia and insulin resistance in NAFLD mice

**Figure 2.**
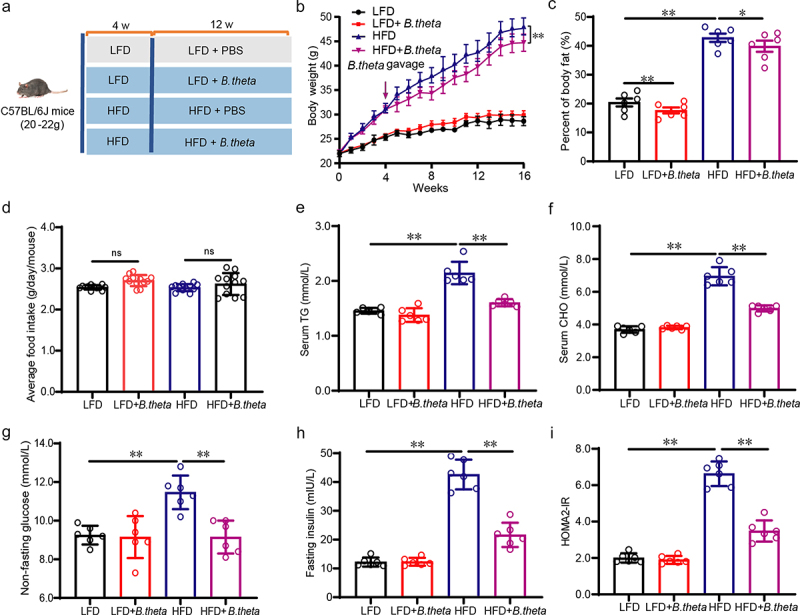
*B. theta* alleviates hyperlipidemia and insulin resistance in NAFLD mice. (a) Animal experimental schedule. (b) Body weight. (c) Body fat. (d) Average food intake per week. (e) Serum TG. (f) Serum CHO. (g) Non-fasting blood glucose. (h) Fasting insulin. (i) HOMA2-IR index. Data are presented as means with SD. *n* = 6, *p < .05 and **p < .01 *vs*. model or control group. ns, not significant.

To examine the causality between lipid metabolism dysfunction in NAFLD and the abundance of *B. theta*, we utilized the HFD-induced obesity mice model to further validate the role of *B. theta*. Male mice were initially treated with a low-fat diet (LFD) or HFD for 4 weeks, followed by continuous treatment with LFD/HFD alone or with *B. theta* gavage three times per week for 12 weeks (Figure 2a). From the first week of treatment, the mice in the *B. theta*-treated HFD group showed a reduced rate of body weight gain, which was significantly lower than that of the HFD group after 12 weeks of treatment (Figure 2b). This result is consistent with previous reports that *B. theta* could reduce body weight in obese mice.^14^ Additionally, *B. theta* treatment led to a slight decrease in body fat percentage in both LFD and HFD-treated mice (Figure 2c). No significant differences were observed in the food intake in both LFD and HFD groups after *B. theta* treatment (Figure 2d), indicating its safety for long-term oral consumption. Moreover, the HFD elevated serum TG and CHO, while supplementation with *B. theta* remarkably reduced them in the HFD but not LFD-treated mice (Figure 2e,f). Furthermore, the HFD increased non-fasting blood glucose (Figure 2g) and fasting insulin (Figure 2h) levels, and the calculated HOMA2-IR index (Figure 2i), whereas *B. theta* effectively reversed these parameters specifically in the HFD-treated group without affecting the LFD-treated group (Figure 2g–i). These results highlight the alleviation of HFD-induced weight gain, hyperlipidemia, and insulin resistance in mice through oral intake of *B. theta* strains without any discernible side effects.

### B. theta treatment prevents hepatic steatohepatitis and liver injury in NAFLD mice

**Figure 3.**
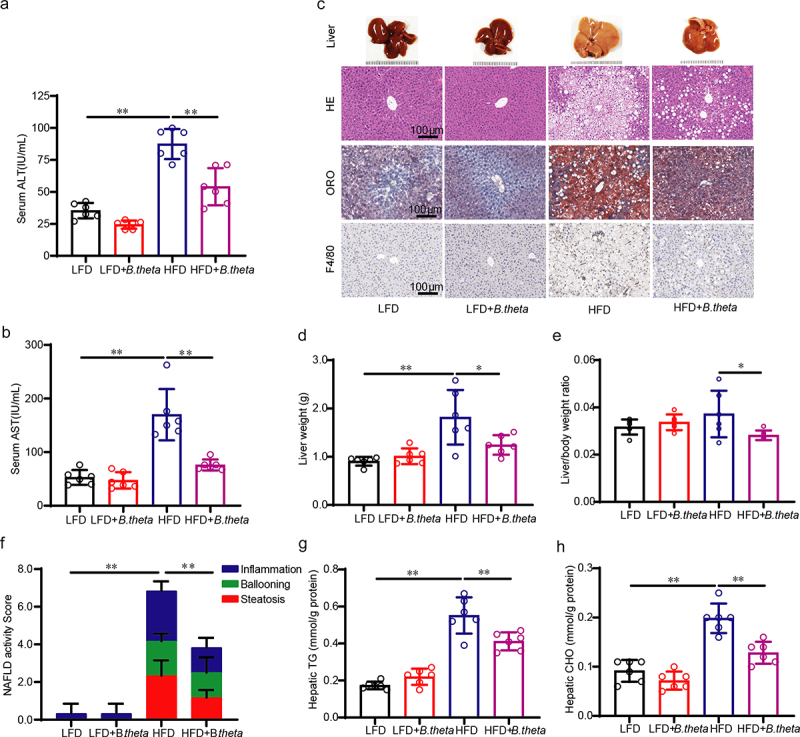
*B. theta* treatment prevents hepatic steatohepatitis and liver injury in NAFLD mice. (a) Serum ALT. (b) Serum AST. (c) Representative liver appearance, H&E staining, oil red O (ORO) staining, and F4/80 immunohistochemical stains of liver sections. (d) Liver weight. (e) Liver index; (f) quantification of NAFLD activity score (NAS), including steatosis, ballooning, and inflammation grade. (g) Liver TG. (h) Liver CHO. The data are presented as means with SD; *n* = 6, *p < .05 and **p < .01 *vs*. model or control group. ns, not significant.

Excessive dietary fat deposition in the liver leads to hepatic steatosis, which, in turn, triggers oxidative stress and inflammatory response in hepatocytes.^17,18^ HFD significantly increased the levels of serum ALT (Figure 3a) and AST (Figure 3b) in mice, suggesting liver injury, whereas treatment with *B. theta* effectively reversed the liver injury. HFD also induced fat accumulation (Figure 3c, H&E and ORO staining) and increased liver weight (Figure 3d), while treatment with *B. theta* alleviated these phenotypes, as well as reducing the liver index (Figure 3e) in the HFD-treated mice, with no significant effect for LFD-fed mice (Figure 3c–e). The liver histopathology of the HFD-fed mice exhibited distorted hepatic lobules, with intracytoplasmic lipid droplets accounting for more than 50% of the field of view, accompanied by inflammatory cell infiltration and ballooning (Figure 3c,f). Remarkably, administration of *B. theta* showed significant improvement in the disrupted hepatic histopathology, evidenced by the quantification of the NAFLD activity score and a reduction in the number of F4/80-positive cells in mice fed with HFD (Figure 3c,f). The lipid-lowering effect of *B. theta* was further demonstrated by decreased hepatic TG (Figure 3g) and CHO (Figure 3h) levels in the HFD-fed mice following treatment, while no such effect was observed in the LFD group. These findings further suggested that *B. theta* treatment might effectively prevent hepatic steatohepatitis and liver injury in NAFLD mice without causing significant toxicity.

### B. theta treatment modulates the gut microbial composition in NAFLD mice

**Figure 4.**
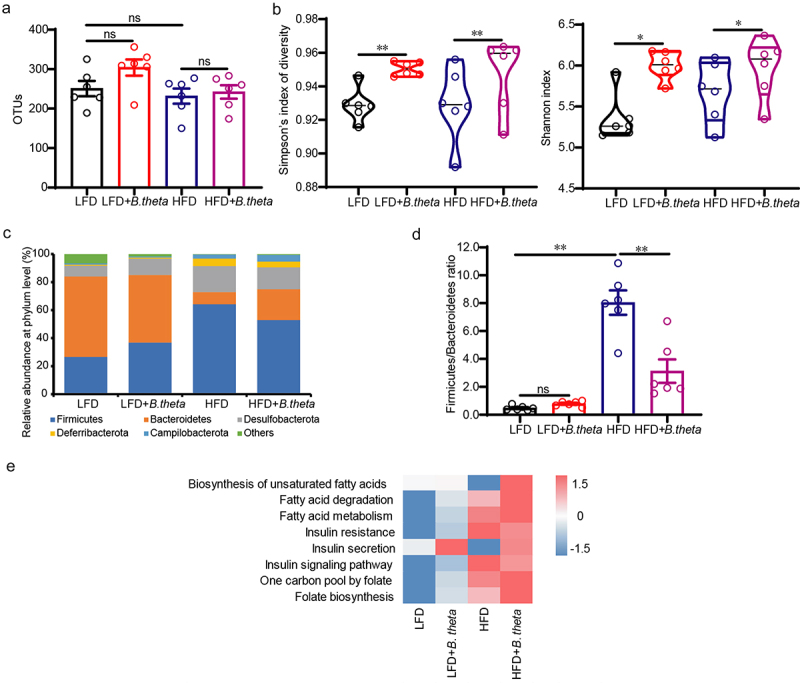
*B. theta* treatment modulates the gut microbial composition in NAFLD mice. The gut microbial composition was obtained through 16s-RNA-amplicon sequencing. (a) Plots of operational taxonomic units (OTUs). (b) Changes in microbiota diversity presented by the Simpson’s index of diversity and Shannon index. (c) Relative abundance of bacteria at the phylum level. (d) The ratio of firmicutes to Bacteroidetes. (e) Prediction of lipid metabolism-related microbiome function based on KEGG database. The data are presented as means with SD; *n* = 6, *p < .05 and **p < .01 *vs*. model or control group. ns, not significant.

To elucidate the underlying mechanisms of *B. theta* in improving hepatic steatohepatitis, we investigated its effects on the gut microbial composition in mice using 16s-RNA-amplicon sequencing. Although the differences in operational taxonomic units (OTUs) were not statistically significant (Figure 4a), *B. theta* treatment significantly increased microbiota diversity, as indicated by higher Simpson’s index of diversity and Shannon index in both LFD and HFD-fed mice (Figure 4b). The ratio of Firmicutes to Bacteroidetes (F/B ratio) is widely accepted to have an important influence on intestinal homeostasis, with an increased F/B ratio often associated with obesity.^19^ At the phylum level, Firmicutes and Bacteroidetes were found to be the dominant bacteria (Figure 4c), and the *B. theta* treatment exhibited a decreased F/B ratio in HFD but not LFD-fed mice (Figure 4c,d), suggesting the potential roles of *B. theta* in affecting intestinal homeostasis and obesity.^19^ KEGG pathway analysis revealed an upregulation in the biosynthesis of folate and unsaturated fatty acids (Figure 4e), which might potentially contribute to the alleviation of obesity, fatty acid metabolism dysfunction, and insulin resistance associated with HFD-induced hepatic steatohepatitis. These results indicated that *B. theta* administration induces changes in gut microbial composition, which may have beneficial effects on alleviating HFD-induced hepatic steatohepatitis.

### B. theta treatment enhances gut folate biosynthesis and modulates hepatic folate metabolism in NAFLD mice

**Figure 5.**
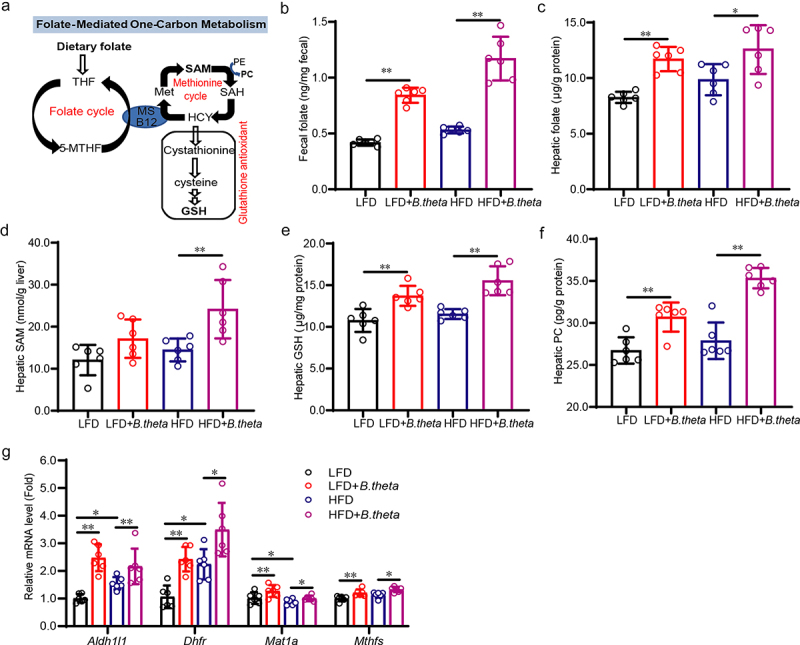
*B. theta* treatment enhances gut folate biosynthesis and modulates hepatic folate metabolism in NAFLD mice. (a) Schematic illustrates a network of folate-mediated one-carbon metabolism. (b,c) total folate level in mouse fecal (b) and liver (c). (d-f) SAM (d), GSH (e), and PC (f) in the mouse liver. (g) mRNAs related to folate-mediated one-carbon metabolism. The data are presented as means with SD; *n* = 6, *p < .05 and **p < .01 *vs*. model or control group. ns, not significant. GSH, glutathione; met, methionine; SAM, S-adenosylmethionine; SAH, S-adenosylhomocysteine; HCY, homocysteine; PE, phosphatidylethanolamine; PC, phosphatidylcholine; MS, vitamin B12-dependent methionine synthase; 5-MTHF, 5-methyltetrahydrofolate.

Folate and folate-mediated one-carbon metabolism (Figure 5a) were widely reported to reduce metabolic abnormalities associated with NAFLD.^20,21^ Based on the enhanced folate biosynthesis and one carbon pool by *B. theta* treatment using KEGG pathways analysis, we thus investigate whether the mechanism and causative effects of the enriched *B. theta* for hepatic steatohepatitis are related to folate metabolism. *B. theta* significantly increased total folate levels in mouse fecal (Figure 5b) and liver (Figure 5c), indicating an enhancement of folate biosynthesis. *B. theta* treatment increased the levels of S-adenosylmethionine (SAM) (Figure 5d), glutathione (GSH) (Figure 5e), and phosphatidylcholine (PC) (Figure 5f), implying activation of folate-mediated one-carbon metabolism in the liver.^21^ The relationship was further confirmed by the higher mRNA levels of folate-mediated one-carbon metabolism-related genes, including *Pemt*, *Aldh1l1*, *Dhfr*, and *Mat1a* (Figure 5g). These findings demonstrated that *B. theta* supplementation might enhance gut folate biosynthesis and modulate hepatic folate metabolism in HFD-fed mice, thereby alleviating metabolic dysfunction associated with NAFLD.

### B. theta regulates the unsaturated fatty acid in NAFLD mice

**Figure 6.**
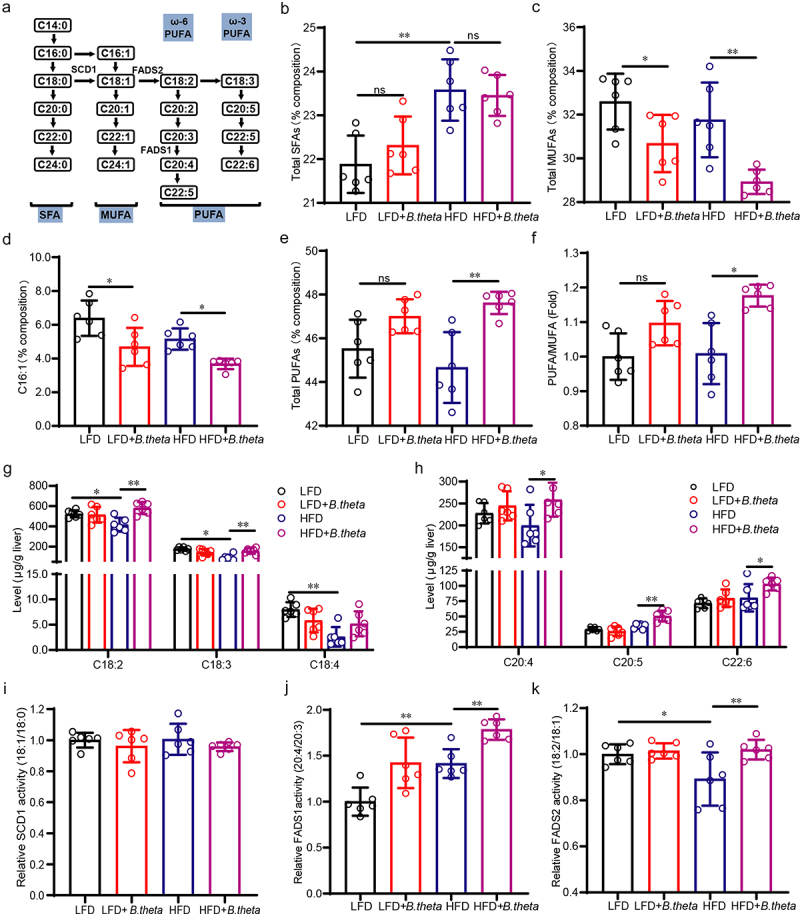
*B. theta* regulates the unsaturated fatty acid in NAFLD mice. (a) Fatty acid composition and the elongation and desaturation process. (b-e) percent composition of total SFAs (b), total MUFAs (c), C16:1 fatty acid (d), and total PUFAs (e). (f) PUFA/MUFA ratio. (g,h) the contents of C18:2, C18:3, C18:4, C20:4, C20:5, and C22:6 PUFAs; (i-k) desaturase activity of stearoyl-CoA desaturase 1 (SCD1), fatty acid desaturase 1 (FADS1) and FADS2. The data are presented as means with SD; *n* = 6, *p < .05 and **p < .01 *vs*. model or control group. ns, not significant.

Multiple studies have suggested that saturated fatty acids (SFAs) and monounsaturated fatty acids (MUFAs) are positively correlated with the severity of liver steatosis, while polyunsaturated fatty acids (PUFAs) and the PUFA/MUFA ratio are negatively correlated with it.^22–25^ In light of the evidence by gut microbial sequencing indicating enhanced biosynthesis of unsaturated fatty acids by *B. theta*, we further investigated the fatty acid composition in the liver (Figure 6a) using the LC-MS/MS method and evaluated the specific fatty acids contributing to the protective role of *B. theta* in NAFLD mice. The results indicated that HFD increased the composition of total SFAs compared with that in the low-fat diet (LFD)-treated group (Figure 6b), which aligned with previous studies.^25^ However, treatment with *B. theta* did not have any impact on total SFAs either in LFD and HFD mice (Figure 6b), suggesting that its effects are independent of SFAs. The total MUFAs were decreased after *B. theta* treatment in both LFD and HFD-fed mice (Figure 6c), primarily due to the decreased levels of palmitoleic/7-hexadecenoic acid (C16:1) (Figure 6d), which suggested the C16:1 might participant the progression of hepatic steatosis and the therapeutic effect *by B. theta*. Importantly, we detected a slight decrease in total PUFAs in HFD-fed model mice and a significant increase after *B. theta* treatment (Figure 6e), resulting in an increased PUFA/MUFA ratio (Figure 6f), two crucial indicators for alleviating NAFLD.^25,26^ The increased composition of PUFAs induced by *B. theta* mainly consisted of C18:2, C18:3, C18:4, C20:4, C20:5, and C22:6, making up more than 80% of the PUFAs (Figure 6g–h). Among them, ω-3 PUFAs (18:3, 20:5, 22:6), which have shown beneficial effects on glycolipid metabolism dysfunction in animal experiments and clinics, are expected to become a new target drug for treating fatty liver.^22,26^ In parallel, *B. theta* did not affect the activity of stearoyl-CoA desaturase 1 (SCD1) (Figure 6i) but significantly increased the activity of fatty acid desaturase 1 (FADS1) (Figure 6j) and FADS2 (Figure 6k), which is consistent with the increased levels of C20:4 and C18:2 after *B. theta* treatment (Figure 6g,h). The multifaceted modulation of PUFAs in the liver of mice mediated by *B. theta* indicates that this is a key mechanism underlying the alleviation of NAFLD. Hence, these findings align with the predictions made by gut microbial sequencing and emphasize the relevance of fatty acid composition changes, specifically the elevated levels of PUFAs induced by *B. theta*, in contributing to its therapeutic role in NAFLD.

## Discussion

**Figure 7.**
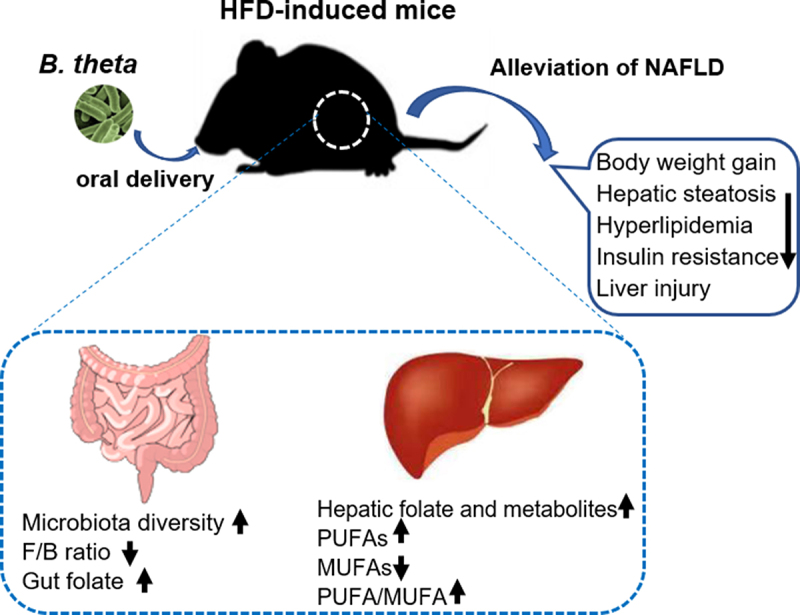
*Bacteroides thetaiotaomicron* (*B. theta*) administration alleviates nonalcoholic fatty liver disease (NAFLD) in HFD-induced mice through multiple mechanisms. A high-fat diet-induced fatty liver in mice accompanied by dysbiosis of gut microbiota and its metabolites. *B. theta* administration resulted in several beneficial effects, as indicated by the following changes: 1) increase in microbiota diversity and decrease in Firmicutes/Bacteroidetes (F/B) ratio; 2) enhancement of gut folate biosynthesis and hepatic folate metabolism; 3) increase in hepatic polyunsaturated fatty acids (PUFAs) and decrease in monounsaturated fatty acids (MUFAs) levels, with decreased PUFA/MUFA ratio. These multiple mechanisms collectively contribute to the therapeutic effects of *B. theta* on the development of NAFLD.

As was widely discussed, the alterations of specific intestinal microbiota could either drive or obstruct the progression of NAFLD.^27,28^ Therefore, there is a need to characterize the gut microbiota in metabolic dysfunction and identify potential microbial targets for therapeutic interventions. In this study, we observed a positive correlation between the abundance of *B. theta* and the improvement of lipid metabolism dysfunction in the early and end-stage of NAFLD. Further study elucidated that *B. theta* administration decreased hyperlipidemia and insulin resistance, while protecting against hepatic steatohepatitis and liver injury in mice with NAFLD. Mechanistically, *B. theta* modulated gut microbiota composition, promoted gut-liver folate metabolism, and regulated hepatic unsaturated fatty acids in NAFLD mice, collectively contributing to the alleviation of hepatic metabolic dysfunction (Figure 7). The findings highlight the importance of gut microbiota modulation, specifically using *B. theta*, as a promising avenue for treating NAFLD and related metabolic disorders.

*Bacteroides thetaiotaomicron*, a representative species of Bacteroides, is commonly found in the intestines of humans and rodents, with a remarkable ability to acquire and degrade plant polysaccharides and regulate immunity and energy metabolism.^12,29,30^ We previously reported that berberine might alleviate NASH partially by regulating gut microbiota.^11^ This study identified *B. theta* as a potential probiotic for treating NAFLD induced by a WD/CCl_4_/DEN combination treatment. This finding was consistent with our deep analysis results in an early NASH mouse model induced by a WD/CCl_4_ treatment.^11^ Though several studies have also reported the role of BBR in regulating gut microbiota,^31,32^ the upregulation for the *B. theta* in different stages of NAFLD upon berberine treatment was first observed in the current work. However, we found that *B. theta* was only associated with improving metabolic disorders but not fibrosis. Therefore, we further investigated the role of *B. theta* in the HFD-induced obese mice, which exhibit metabolic syndromes without fibrosis and are known to have altered microbial communities.^33,34^ Previous studies have shown that gavage with *B. theta* can protect mice against adiposity.^14,35^ Similarly, we found that *B. theta* decreased hyperlipidemia and insulin resistance and prevented hepatic steatohepatitis and liver injury in HFD-fed mice. Importantly, we observed that *B. theta* did not affect food intake, body weight, and hepatic histopathology in LFD-fed mice, which indicated its safety. However, it is worth noting that *B. theta* has also been reported to promote diet-induced obesity in antibiotic-treated mice.^27^ We hypothesize that the intestinal microenvironment may be disrupted in mice treated with antibiotics or those that are germ-free. This disruption could potentially impact the effects of B. theta, as its anti-obesity properties may be influenced by the presence of a specific intestinal microenvironment or its interaction with other microbial species or bacteria-host interplay.^12^ As evidence, we found that gavage with *B. theta* resulted in increased microbiota diversity in both low-fat and high-fat diet-fed mice (Figure 4b), as well as a decreased Firmicutes/Bacteroidetes (F/B) ratio in HFD-fed mice (Figure 4c,d), which is typically associated with obesity. Moreover, some bacteria have been reported to interact with or enhance the effects of *B. theta*, while *B. theta* itself may also influence their abundance.^5^ For example, *A. muciniphila*, which has been reported to counteract adiposity in HFD mice,^36^ was found to be increased after *B. theta* gavage in our study (Data not shown). Further studies are warranted to elucidate how *B. theta*, in conjunction with other microbial species, modulates metabolism.

Previous studies have highlighted various mechanisms through which *B. theta* improves fatty liver disease. For instance, *B.* *theta* has been shown to decrease body weight gain and adiposity and regulate lipid metabolism-related gene expression in white adipose tissue of mice fed with high-fat diets.^14^ Additionally, *B. theta* could ameliorate hepatic steatosis in experimental alcohol-related liver disease through a restored mucosal barrier and reduced LPS translocation.^13^ Moreover, *B. theta*-derived sphingolipids have been observed to be transferred from the microbiome to the colon and liver, improving diet-induced hepatic steatosis.^30^ In line with these previous findings, we further investigated the new functions and mechanisms of *B. theta* in our study. We demonstrated that *B. theta* regulated gut microbiota composition, increased gut folate biosynthesis, and modulated liver folate metabolism. Folate supplementation in rodents has been widely reported to reduce metabolic abnormalities associated with NAFLD, including steatosis, inflammation, oxidative stress, insulin resistance, and hyperglycemia.^20,21,37^ We also examined the effect of *B. theta* on hepatic S-adenosylmethionine (SAM) levels and downstream metabolites, such as phosphatidylcholine (PC) and glutathione (GSH) synthesis. SAM is involved in key metabolic pathways, including transmethylation and transsulfuration, and is crucial for the synthesis of PC, an essential component for very low-density lipoprotein assembly and lipid export. SAM also participates in the synthesis of cysteine, a precursor for taurine and GSH, which play roles in countering hepatic oxidative stress.^21^ Remarkably, our study has made the novel discovery that *B. theta* supplementation in HFD-fed obese mice resulted in a decrease in MUFAs and an increase in PUFAs in mice liver, while SFAs remained unaffected. The potential benefits of these changes by *B. theta* are consistent with previous conclusions indicating that increased levels of SFAs and MUFAs accelerated the severity of liver steatosis, whereas higher levels of PUFAs and a higher PUFA/MUFA ratio are negatively correlated with the NAFLD progression.^22–25^ Hence, these results further supported the intricate mechanisms by which *B. theta* may treat NAFLD.

In summary, our study expands on the knowledge of *B. theta*‘s functions and mechanisms in the context of fatty liver disease. By regulating the gut microbiota structure, increasing gut folate synthesis, modulating gut-liver folate metabolism, and enhancing hepatic unsaturated fatty acid levels, *B. theta* might exhibit promising therapeutic potential for NAFLD. Our discovery also provides potential avenues for future research and intervention strategies targeting the gut microbiota in treating the metabolic dysfunction in NAFLD.

## Materials and methods

### Preparation of bacteria strains

The *Bacteroides thetaiotaomicron* (*B. theta*) strain ATCC 29,148 was derived from ATCC and cultured on blood agar at 37°C under strictly anaerobic conditions. Cultures were centrifuged at 2500 g for 10 minutes, washed twice with sterile phosphate-buffered saline (PBS), and re-suspended in sterile PBS containing 10% glycerol to achieve a concentration of 1 × 10^9^ colony-forming units (CFUs) per milliliter. The bacterial suspension was stored at −80°C for subsequent use. A viability confirmation was performed by culture to guarantee that *B. theta* grew well. For *in vivo* experiments, live *B. theta* was administered to C57BL/6J mice *via* oral gavage three times per week for a total duration of 12 weeks. Each dose consisted of 1 × 10^8^ CFUs of *B. theta* suspended in 0.1 mL of PBS per mouse.

### Animal experiments

Male C57BL/6J mice were obtained from SPF (Beijing) Biotechnology Co., Ltd. and were housed in a 12-hour light/dark light cycle with ad libitum access to water and food. As previously described,^38^ the mice were subjected to a western diet/carbon tetrachloride/dimethylnitrosamine (WD/CCl_4_/DEN)-induced model (Figure 1a, up). Briefly, the mice (25.0–27.0 g) were fed a western diet (TP26300122, Trophic Animal Feed High-Tech Co., Ltd. China) along with high-sugar drinking water containing 23.1 g/L d-fructose and 18.9 g/L d-glucose. They were intraperitoneally injected with 0.2 mL/kg CCl_4_ in corn oil once a week. After 4 weeks of induction, the mice were continuously exposed to the WD/CCl_4_ regimen and treated with or without berberine (BBR) in food (equivalently 200 mg/kg/day by gavage) for an additional 20 weeks. At the 10-week mark of BBR treatment, a single intraperitoneal injection of 100 mg/kg DEN was administered, and the dose of CCl_4_ was increased from 0.2 mL/kg to 0.5 mL/kg. Similarly, WD/CCl_4_-induced mouse model with a 4-week pretreatment of western diet/carbon tetrachloride (WD/CCl_4_) and subsequent 8 weeks therapy of 200 mg/kg/day BBR in food (Figure 1a, down), was also utilized as we previously reported.^11^ For the animal experiments involving *B. theta* treatment, 24 mice (20.0–22.0 g) were randomly assigned to four groups: LFD+PBS (10% low-fat diet with 100 µL PBS gavage per mouse), LFD+*B. theta* (10% low-fat diet with 100 µL *B. theta* gavage per mouse), HFD+PBS (60% high-fat diet with PBS gavage), and HFD+*B. theta* (60% high-fat diet with *B. theta* gavage). The mice were treated with LFD or HFD for 4 weeks, followed by *B. theta* or PBS gavage for 12 weeks. Food intake and body weight were recorded weekly. The mouse fat mass was measured using an NMR body composition analyzer (QMR06-090 H, NIUMAG, China) one day before the end of the experiment. Fasting and non-fasting blood glucose levels were measured *via* the tail vein using a blood glucose meter (ACCU-CHECK Performa, Roche, Switzerland). At the end of the experiment, the mice were sacrificed, and blood samples were collected. Intralobular pieces of the liver were quickly frozen in liquid nitrogen for liver biochemistry and subsequent mechanism studies. For histological analyses, liver slices were fixed with 4% paraformaldehyde (Servicebio, #G1101). This study was approved by the Institutional Animal Care and Use Committee of the Institute of Medicinal Biotechnology, Chinese Academy of Medical Sciences (SYXK(Jing) 2017–0023), and animal experiments were conducted following the National Guidelines for Housing and Care of Laboratory Animals.

### Analysis of biochemical parameters

The collected blood samples were centrifuged at 2500 g for 10 minutes, and the serum was collected. Commercial assay kits from Nanjing Jiancheng Bioengineering Institute (Nanjing, China) were used to measure the serum levels of ALT (C009-2-1), AST (C010-2-1), TG (A110-1-1-TG), and CHO (A111-1-1). Serum insulin was measured using a mouse insulin ELISA kit (SEKM-0141, Solarbio). Insulin resistance was calculated using a homeostasis model assessment‐2 (HOMA2) index through an online-based calculator on the Diabetes Trials Unit of the University of Oxford website (https://www.dtu.ox.ac.uk/homacalculator/). Liver biochemistry analysis was performed by homogenizing the mouse liver, and the levels of hepatic TG and CHO were measured using assay kits according to the manufacturer’s instructions. Commercial kits were also used to measure folate (FA, E-EL-0009c, Elabscience), S-adenosylmethionine (SAM, FY-EU12032, Wuhan Feiyue Biotechnology Co., Ltd), glutathione (GSH, BC1175, Solarbio), and phosphatidylcholine (PC, KS13966, Shanghai Keshun Science and Technology Co., Ltd.). The liver homogenates were extracted using the corresponding lysis buffer with protease inhibitor cocktail (C0001, Targetmol) and quantified using the BCA protein assay kit (23225, Thermo Scientific, New York, NY, USA).

### Histological analysis

The fixed liver tissues were stained with hematoxylin and eosin (H&E) staining to evaluate liver steatosis, ballooning, and inflammation. The NAFLD classifications were assessed by two experts blinded to the experimental groups according to the NAFLD activity score (NAS) criteria, which incorporates scores for steatosis (0–3), hepatocellular ballooning (0–2), and lobular inflammation (0–3).^39^ Similarly, the Masson trichrome staining was conducted to evaluate the fibrosis stage (0–4). Steatosis was further confirmed by Oil Red O (ORO) staining in frozen sections. Immunohistochemical (IHC) staining of F4/80 (GB113373, Servicebio, China), a macrophage lineage marker,^40^ was performed to further evaluate the inflammation in the liver.

### Intestinal microbiological analysis

Five or six mice in each group were selected to perform gut microbiological analysis using 16S rRNA gene sequencing by Oebiotech (Shanghai, China). Briefly, genomic DNA was isolated from samples using the DNeasy PowerSoil Kit (QIAGEN) and amplified using primers specific for the V3-V4 region of 16S rRNA (343F–5ʹ TACGGRAGGCAGCAG 3ʹ and 798 R–5ʹAGGGTATCTAATCCT3ʹ). The amplification products were checked by 1% agarose gel electrophoresis, purified using AMPure XP beads (Agencourt), and subjected to another round of PCR, as described earlier.^11^ The final amplicon was quantified using the Qubit quantification system (Life Technologies) and purified again. Equal amounts of purified amplicons were pooled for subsequent sequencing using the Illumina MiSeq System (Illumina Inc., San Diego, CA, USA). The raw FASTQ sequencing files were preprocessed for bioinformatics analysis using QIIME software (version 1.8.0). The clean reads were processed to remove primer sequences and cluster them into operational taxonomic units (OTUs) using V search software (version 2.4.2) with a 97% similarity cutoff. Based on the OTU counts, the community structure and alpha diversity (presented as the Simpson’s index of diversity and Shannon index) were analyzed to assess the composition of the sample community. Functional inference associated with metabolic regulation was analyzed using the Kyoto Encyclopedia of Genes and Genomes (KEGG) pathways.

### Liquid chromatography-tandem mass spectrometry (LC-MS/MS) quantitative analysis of long-chain fatty acid

Freshly collected liver samples were frozen in liquid nitrogen and stored at −80°C until further analysis. The determination of long-chain fatty acids, including saturated fatty acids (SFAs), monounsaturated fatty acids (MUFAs), and polyunsaturated fatty acids (PUFAs), were conducted by the Beijing Bio-Tech Pack Technology Company Ltd. In brief, a 50 mg liver sample was suspended in 1 mL Isopropyl alcohol: acetonitrile (1:1) and then vortexed and centrifugation at 12,000 g for 10 minutes. The concentrations of fatty acids in supernatants were detected by LC-MS/MS, with fatty acids (19:0) as the internal standard. Chromatography was performed on Waters UPLC BEH C8 column (2.1 × 100 mm, 1.7 µm) with a column temperature of 55°C. Phase A (acetonitrile: water = 1:10, 0.1% acetic acid, 1 mM ammonium acetate) and phase B (isopropyl alcohol: acetonitrile = 1:1) were used as the mobile phase for gradient elution, with a flow rate of 0.26 mL/minute and a sample loading volume of 5 μL. The Waters XEVO TQ-S Micro mass spectrometry system was used for mass spectrometry analysis in negative ion mode ESI. Other parameters were as follows: Ion source voltage −2 kV, temperature 150°C, desolvation temperature 500°C, desolvation gas flow rate 1000 L/hour; The micro-hole voltage 10.0 V and the gas flow rate 150 L/hour. The peak area of targeted data was calculated using TargetLynx quantitative software.

### Activity index of desaturase

The activity of stearoyl-CoA desaturase 1 (SCD1), fatty acid desaturase 1 (FADS1), and FADS2 was estimated by assessing the product-to-precursor ratios, specifically C18:1/C18:0 for SCD1, C20:4/C20:3 for FADS1, and C18:2/C18:1 for FADS2. This approach has been extensively utilized in prior studies.^25^

### RNA extraction and real-time quantitative PCR

Total RNA in liver tissues was extracted using RaPure total RNA kit (R4011, Magen) according to the manufacturer’s protocol. The mRNA levels of *Pemt*, *Aldh1l1*, *Dhfr*, and *Mat1a* were amplified with specific primers (Table 1) and quantified with one-step real-time quantitative reverse transcript PCR (qRT-PCR) using HiScript II One Step qRT-PCR SYBR Green Kit (Q221–01, Vazyme) as previously used.^38^ The relative mRNA amounts were calculated by the comparative Ct method after normalizing against the amount of the internal control mRNA level of glyceraldehyde 3-phosphate dehydrogenase (*Gapdh*).

**Table 1.** Sequence of primers.

Genes	Forward primer (5’→3’)	Reverse primer (5’→3’)
*Aldh1l1*	CAGGAGGTTTACTGCCAGCTA	CACGTTGAGTTCTGCACCCA
*Dhfr*	CGCTCAGGAACGAGTTCAAGT	TGCCAATTCCGGTTGTTCAATA
*Mat1a*	GTGCTGGATGCTCACCTCAAG	CCACCCGCTGGTAATCAACC
*Mthfs*	ACCAATTCCAGAGCAATCACAT	CTCCTCCCGAACATCTCCCT

### Statistical analyses

The data were presented as mean ± standard deviation (SD) or representative figures. The data were analyzed by analysis of variance (ANOVA) followed by Student-Newman-Keuls (SNK) post hoc tests using GraphPad Prism 8 software. Kruskal-Wallis H test or Mann-Whitney U test were used for the nonparametric test. The threshold for statistical significance was set at *p < 0.05 or ** *p* < 0.01.

## Data Availability

The data that support the findings of this study are available in the article or from the corresponding author upon reasonable request.
